# Direct observation systems for child behavior assessment in early childhood education: a systematic literature review

**DOI:** 10.1007/s44192-025-00139-z

**Published:** 2025-02-24

**Authors:** Maha Al-Hendawi, Esraa Hussein, Sughra Darwish

**Affiliations:** Department of Psychological Sciences, College of Education, Qatar University, P.O. Box 2713, Doha, Qatar

**Keywords:** Child behavior, Early childhood education, Direct observation systems, Behavioral disorders

## Abstract

This systematic literature review critically assessed the use of direct observation systems (DOSs) in early childhood educational research, highlighting their role in the objective and systematic evaluation of child behavior and classroom dynamics. Our comprehensive analysis distinguishes between standardized and non-standardized observation systems regarding their applications, target groups, and capabilities for assessing diverse behavioral constructs in early childhood education settings. We synthesized data from 88 empirical studies, including standardized (47 studies) and non-standardized (41 studies) approaches. While the standardized DOS was defined as that which was consistently replicated across multiple research projects, the non-standardized DOS was tailored to the unique requirements of individual studies. Our findings suggest that while the standardized DOS offers a reliable method for generalized behavioral assessment, the non-standardized DOS is also widely and effectively used because it provides the flexibility required for more targeted behavioral evaluations. The Classroom Assessment Scoring System (CLASS) and the Individualized Classroom Assessment Scoring System (inCLASS) represent commonly used standardized tools, while non-standardized systems demonstrate particular strength in context-specific applications. The majority of the research was situated within pre-elementary contexts, with standardized studies having a median sample size of 158 compared to 136 non-standardized studies. Gender representation across the studies was notably equitable. A significant portion of related research has focused on populations at-risk of or diagnosed with emotional/behavioral disorders (EBDs) and attention-deficit/hyperactivity disorder (ADHD), accounting for 59.6% of standardized studies and 80.5% of non-standardized studies. DOS predominantly addressed emotional regulation (42%) and externalizing behaviors (21%).

**Supplementary Information:**

The online version contains supplementary material available at 10.1007/s44192-025-00139-z.

## Introduction

Direct observation (DO) has emerged as a crucial tool in educational research, particularly for conducting functional behavioral assessments over time. This methodology is invaluable for identifying the unique needs and behaviors of children, highlighting both their strengths and areas requiring intervention [4, 49]. The robustness of DO data has found applications in various aspects of education, from examining teacher-student relationships to diagnosing emotional and behavioral disorders (EBDs) in children [1, 72].

Understanding classroom behavior and teacher-student interactions is a complex endeavor, especially given the varied behaviors exhibited by students [4, 23]. The inherently diverse spectrum of student behaviors poses a challenge in selecting the most appropriate direct observation system (DOS). While overt behaviors, such as disruptions, are more easily recorded, subtler internalizing behaviors, such as anxiety, which have a significant impact on academic and social performance, are more difficult to identify [1, 6, 7, 47]. This makes the choice of an optimal DOS a complex task given the myriad of available options, from simple narrative methods to complex technology-driven systems.

In the field of behavioral analysis, it is essential to distinguish between two fundamental assessment approaches: descriptive and indirect. Descriptive assessment, through systematic direct observation, involves precise measurement of both target behaviors and relevant environmental events via repeated observation and coding [4, 51]. This method provides empirical, time-linked data of behavior-environment interactions, offering a level of insight that is not possible through other means [59]. Conversely, indirect methods, including interviews and rating scales, serve distinct purposes by gathering informant perspectives on behavioral typographies, contextual patterns, and intervention responses [46]. Each approach has specific strengths and limitations that influence its utility and optimal timing in behavioral assessments.

Within this intricate landscape, researchers commonly use either standardized DOS (sDOS) or customized/non-standardized DOS (nsDOS), depending on their specific research goals. While sDOS systems offer consistency, nsDOS systems allow for a customized focus on specific research questions and distinct student demographics. Regardless of the chosen system, a rigorous evaluation of its reliability and psychometric integrity is vital for meaningful data collection and interpretation [13].

Building on the methodological rigor of descriptive assessments, several standardized direct observation systems have been developed, particularly in early childhood education settings. These systems employ structured protocols, often using checklists, and are administered by trained professionals who conduct repeated observations to ensure consistent and in-depth data collection [43]. A critical aspect of these systems is the use and determination of interobserver agreement (IOA), which is essential for establishing the reliability and validity of behavioral data collection[77]. The consideration of IOA is a key metric in analyzing the effectiveness of tools for assessing student behavior.

The application of DOS has broadened the scope of real-time observational research, revealing both typical and unexpected behaviors in natural settings [18]. This granular focus on daily behavior reveals distinctions that might otherwise be overlooked [19]. The field has also embraced technological advancements such as video-based observational studies. These studies utilized specialized coding systems to capture and analyze intricate behavioral patterns, social interaction, and academic engagement, thereby enriching our understanding of classroom dynamics [61, 69].

In recent years, the use of DOS in early childhood education has expanded significantly, with researchers and practitioners employing both standardized and nonstandardized systems. Standardized DOS, such as the CLASS, inCLASS, and Teacher Coder Observation System (TCOS), provide structured frameworks for assessing various aspects of child behavior and classroom interactions. On the other hand, non-standardized DOS often involves custom-developed observation protocols tailored to specific research questions or contexts.

Although numerous individual studies on DOS exist, there is a notable absence of systematic reviews that offer a comprehensive analysis. This gap is especially significant given the importance of early childhood education, where timely observations and interventions can have a lasting impact on a child's development. A targeted systematic review can fill this void by providing valuable insights into the relative merits and drawbacks of different DOSs. Such a review could clarify the effectiveness of these interventions in monitoring behaviors and facilitating evidence-based interventions in young children, a crucial stage in educational development. This could also help to identify gaps in the current literature, such as discrepancies in DOS applications across different educational settings and age groups. By synthesizing the existing literature on DOS in early childhood education, this review seeks to identify the most commonly used standardized and non-standardized DOS, characterize the types of behaviors assessed using these systems, examine the demographic characteristics of children observed in these studies, highlight patterns in DOS implementation across various educational settings, identify gaps in the current research, and propose future directions for the field.

### DOS in the educational setting

Direct observation (DO) stems from the behavioral theory, which focuses on observable and measurable behaviors and environmental influences [1, 23, 49, 63, 73]. Unlike other methods, such as self-reports, DO highlights observing behaviors in natural settings and quantifies them for optimal objectivity. Over time, several DOS have been developed to enable structured and objective observation. Overall, applied behavior analysis, attachment theory, and ecological systems theory provide complementary lenses for classroom observation tools to capture teacher practices, child behaviors, interaction dynamics, and implications for learning and development. This blend of behavioral, developmental, and ecological perspectives enriches the utility of DOS.

#### sDOS

The sDOS systems refer to structured and validated tools for which manuals specifying administration and coding procedures have been published (Table 1). These systems have prescribed observational categories, codes, and protocols grounded in theory and empirical testing [42, 64]. Standardized tools undergo rigorous psychometric testing to demonstrate strong reliability and validity in assessing target behaviors, interactions, or processes. The administration procedures, coding schemes, and protocols were standardized to maximize interrater reliability across different observers and settings. Prominent sDOS include the CLASS [48, 66], Direct Observation Form (DOF), Behavioral Observation of Students in Schools (BOSS)[66], Multi-Option Obsestem for Experimental Studies (MOOSES) [75], and Revised Edition of the School Observation Coding System (REDSOCS)[44] (features of these instruments are given in Table 1 and Supplementary file: Appendix 1).

**Table 1:** Key features of sDOS

Observation System	Key Features	Psychometrics	References
Behavioral Observation of Students in Schools (BOSS)	Combines interval recording and time sampling; Captures active engagement, passive off-task, disruptions	Interrater reliability: 0.87–0.96; Distinguished normal and ADHD groups	(Shapiro, 2003)
Classroom Assessment Scoring System (CLASS)	Assesses teacher–child interactions and classroom climate; Used for professional development	Internal consistency: 0.79–0.94; Associated with positive interactions and environments	(La Paro et al., 2004)
Individualized Classroom Assessment Scoring System (inCLASS)	Focuses on individual child interactions; Dimensions include engagement with teachers, peers, tasks	Interrater reliability: 0.71–0.95; Related to emotional regulation	(Downer et al., 2010)
Multi-Option Observation System for Experimental Studies (MOOSES)	Highly adaptable system for coding diverse behaviors in research	Reliability: 0.71–0.99; Enables detailed timestamping and analysis	(Tapp et al., 1995)
Minnesota Preschool Affect Checklist, Revised and Shortened (MPAC-R/S)	Categorizes behavioral motivations; Informs function-based interventions	Interrater reliability: 0.89; Validated to behavioral functions	(Susanne A. Denham et al., 2012)
Researcher-Educator Collaboration for Developing Observation Coding Systems (REDSOCS)	Collaborative development of customized tools; Tailored to unique settings	Interrater reliability > 0.80; Flexibility for specific contexts	(Jacobs et al., 2000)
Teacher–Child Interaction Direct Observation System (TCIDOS)	Examines quality and nuances of teacher–child interactions	Convergent validity with CLASS	(Kevin S. Sutherland et al., 2013)

#### nsDOS

Conversely, nsDOSs are customized observation tools or systems that may not have undergone extensive validation or standardization processes (Table 2). These systems are often tailored to specific research questions, settings, or populations and may not have established protocols for coding or interpreting observations [11, 71]. Moreover, nsDOS systems allow increased flexibility in observational coding schemes and procedures. They did not rely on extensively published manuals or prescribed administration protocols, and may have incorporated a mix of observational categories drawn from multiple existing theories, tools, or frameworks based on their needs. Specific observational criteria, codes, and procedures can be adapted to suit unique research questions or classroom settings for a particular study. Reliability testing involves the extensive training of coders in a study-specific observation scheme. While this customizability allows non-standardized approaches to be highly responsive to researchers’ aims, it reduces standardization and comparability with other tools or studies. Examples of nsDOS include the unique adaptations of interval recording, time sampling, and narrative records tailored to individual research objectives[3, 29, 30].

**Table 2:** Key features of nsDOS

Classification	Focus Area	Methodology	Tools Used	Examples
Video-Based Observational Studies	Children's behaviors, social interactions, or academic engagement	Video recordings	Specialized coding systems	(Debra A. Prykanowski et al., 2018; Tina L. Stanton-Chapman et al., 2014)
Social and Emotional Behavior Studies	Social and emotional aspects of children's behaviors	Direct observations, coding systems	Adapted or specialized coding systems	(Sallquist et al., 2012; Wright et al., 2022)
Classroom Behavior and Teacher Interaction Studies	Student behaviors and teacher interactions in the classroom	Direct observations, coding systems	Established observation codes	(Schaffner et al., 2016; Catherine Tucker et al., 2017)
Activity and Task-Based Observational Studies	Specific activities or tasks like playtime, academic tasks, or transitional periods	Time sampling or interval-based methods	Time sampling or interval-based coding systems	(Stutey et al., 2017; Zaghlawan & Ostrosky, 2011)
Reliability and Validity-Focused Studies	Reliability and validity of the observation methods	Multiple observers, interobserver agreement assessments	Cross-validation with other measures	(Benner et al., 2012; Li Luo et al., 2017)

## Methods

### Literature search

A systematic review was conducted across five electronic databases—ERIC, Scopus, PubMed, PsycArticles, and Teacher Reference Center—utilizing the search terms 'observation,' 'behaviors,' and 'early childhood.' The term 'observation' was intentionally broadened to encompass both direct and indirect approaches. 'Early childhood' is defined as birth to 8 years of age, aligning with prevailing definitions in the literature [6, 11].

However, our study focuses specifically on children’s behaviors in educational settings, encompassing the ages of three to eight years, which include structured educational environments, such as preschools, kindergartens, and early elementary grades. The searches of the reference lists further supplemented the database search.

**Fig. 1:**
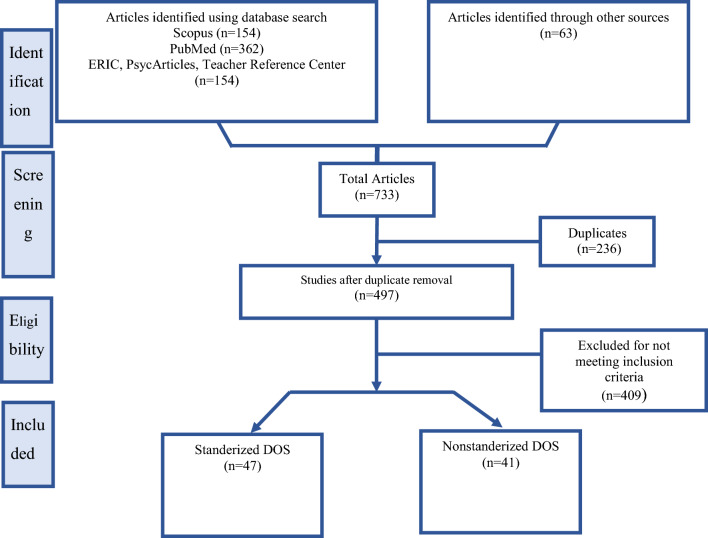
PRISMA study flowchart

The inclusion criteria for this systematic review were to maintain a focused and rigorous scope. The articles were required to discuss or implement DOS in their methodology. For sDOS, publications from all years were considered, capturing the evolution and enduring methodologies that have stood the test of time. In contrast, for nDOS, the review was limited to articles published over the past decade. This decision focused on the most current and emerging trends, capturing innovative methodologies that shaped the future of the field. Given the rapidly evolving nature of non-standardized observation tools, a more contemporary timeframe was deemed appropriate to provide insights into the latest advancements and applications (Fig. 1). By applying PRISMA (Preferred Reporting Items for Systematic Reviews and Meta-Analyses) guidelines (Page et al., 2021), we aimed to enhance the quality, transparency, and reproducibility of our systematic review, ensuring that our findings are robust and reliable. Figure 1 provides a detailed description of the PRISMA flowchart, which provides a clear and comprehensive account of the review process. We have provided a PRISMA flowchart (Fig. 1) in the manuscript, which visually represents the flow of information through the different phases of the review. The flowchart details the number of studies identified, screened, assessed for eligibility, and included in the review, along with the reasons for exclusion at each stage.

To ensure scientific rigor of the review, only peer-reviewed journal articles were included. The review was restricted to articles published in English to maintain consistency of interpretation across studies. The targeted age group was early childhood populations, specifically those ranging from birth to eight years, to align with key developmental and educational milestones. Moreover, studies must be conducted in educational settings such as preschools or elementary schools to focus on educational research and practice.

### Coding procedures and data analysis

Our systematic review adhered to PRISMA guidelines to ensure a thorough and unbiased approach throughout the study. Both authors independently screened the titles and abstracts of all retrieved studies to identify those that met the inclusion criteria. Any disagreements regarding the inclusion of studies were resolved through discussion and consensus to ensure a comprehensive and objective selection process. Data extraction from the included studies was meticulously performed by both authors independently, minimizing errors and discrepancies. Any differences in data extraction were discussed and resolved through mutual agreement, thereby maintaining the accuracy and consistency of the collected data. The risk of bias and the quality of the included studies were independently assessed by each author. In addition, 35% of the studies were randomly selected and cross-examined by the authors to ensure reliability. Inter-rater reliability checks were conducted, achieving an agreement of > 97%. Any discrepancies were resolved through consensus, reducing potential bias and enhancing the accuracy of our review process.

A structured coding worksheet was developed in Microsoft Excel to categorize articles based on these selection criteria. Descriptive statistics were used to summarize the characteristics of the included studies.

## Results

### Pooled analysis: sDOS

In the analyzed studies, the CLASS and inCLASS instruments were the most frequently employed instruments; these instruments were used in 28 studies and accounted for approximately 59.6% of the total research (Table 3). Five studies used the DOF instrument, accounting for approximately 10.6% of the total studies. TCIDOS was employed in six studies, representing approximately 12.8% of the overall research. Other instruments, such as the MPAC-R/S, MOOSES, REDSOCS, and BOSS, were collectively used in eight studies, contributing to approximately 17.0% of the total. The age group most commonly studied was 3–5 years. The gender distribution across the studies was fairly even, with a median percentage of 53.8% boys participating. The clinical classification revealed that 19 studies (40.4%) did not specify any disability or focused on children without any clinical classification. Eleven studies (23.4%) targeted at-risk children and 17 (36.2%) focused on children with EBD or ADHD. In terms of educational settings, the majority of the studies were conducted in pre-elementary settings, represented by 26 studies (55.3% of the total). The elementary settings were the focus of 20 studies, accounting for approximately 42.6% of the research. Only one study (2.1%) did not specify grade level.

**Table 3:** Pooled Data Summary of Studies (N = 47) using sDOS

Category	Description	Data
Number of Participants	Median	158.0
	Interquartile Range (IQR)	20.5—352.5
Gender Distribution (Boys)	Median Percentage	53.8%
	Interquartile Range (IQR)	50.0%—65.8%
Clinical Classification	Not Reported/No disability	19 (40.4%)
	At-risk	11 (23.4%)
	EBD/ADHD	17 (36.2%)
Instruments Used	CLASS and inCLASS	28 (59.6%)
	DOF	5 (10.6%)
	TCIDOS	6 (12.8%)
	Other Instruments (MPAC-R/S, MOOSES, REDSOCS, BOSS)	8 (17.0%)
Grade Level	Other/Not Mentioned	1 (2.1%)
	Pre-Elementary	26 (55.3%)
	Elementary	20 (42.6%)

*ADHD* = *Attention-Deficit Hyperactivity Disorder, BOSS* = *Behavioral Observation of Students in Schools, CLASS* = *Classroom Assessment Scoring System, DOF* = *Direct Observation Form, EBD* = *Emotional and Behavioral Disorders, inCLASS* = *Individualized Classroom Assessment Scoring System, IQR* = *interquartile range, MPAC-R/S* = *Minnesota Preschool Affect Checklist—Revised/Shortened, MOOSES* = *Multi-Option Observation System for Experimental Studies, N* = *total number of participants, REDSOCS* = *Revised Edition of the School Observation Coding System, TCIDOS* = *Teacher–Child Interaction Direct Observation System*

### Pooled analysis: nsDOS

In studies that employed the nsDOS, the median number of participants was 136, with an interquartile range (IQR) ranging from 20 to 450 participants (Table 4). The sex distribution was relatively balanced, with a median percentage of 51.0% of boys participating in the study. The IQR for sex distribution ranged from 43.0% to 81.2%. Regarding clinical classification, eight studies (19.5%) did not specify any disability or focused on children without any clinical classification. A significant proportion (20 studies, 48.8%) targeted at-risk children. Thirteen studies (31.7%) focused on children with EBD, ADHD, or autism spectrum disorder (ASD). Regarding the instruments used, 18 studies (43.9%) employed custom or author-developed observation systems, whereas 23 studies (56.1%) used existing non-standardized systems. The majority of the research was conducted in pre-elementary educational settings and was represented by 30 studies (approximately 73.2% of the total). Eleven studies focus on 11 studies, accounting for approximately 26.8% of the research.

**Table 4:** Summary of Key Features of Studies (N = 41) using nsDOS

Category	Description	Data
Number of Participants	Median	136.0
	Interquartile Range (IQR)	20–450
Gender Distribution (Boys)	Median Percentage	51.0%
	Interquartile Range (IQR)	43.0%–81.2%
Clinical Classification	Not Reported/No disability	8 (19.5%)
	At-risk	20 (48.8%)
	EBD/ADHD/ASD	13 (31.7%)
Instruments Used	Custom/Author-developed	18 (43.9%)
	Existing Non-standardized	23 (56.1%)
Grade Level	Pre-Elementary	30 (73.2%)
	Elementary	11 (26.8%)

*At-risk: Refers to children who are considered to be at a higher risk of developing academic or behavioral problems; EBD/ADHD/ASD: Emotional and Behavioral Disorders / Attention-Deficit/Hyperactivity Disorder/Autism Spectrum Disorder; Elementary: Refers to educational settings that include kindergarten through 5th or 6th grade, IQR: Interquartile Range, Pre-elementary: Refers to educational settings such as preschools and pre-kindergarten*

### Systematic review

#### Key findings from studies using sDOS

We found that the relative distribution of different sDOSs in the scholarly literature is as follows: almost 42% of the studies focused on emotional regulation and social adaptation and 21% focused on externalizing behavior. Emotional regulation accounted for 16%, classroom interactions for 12%, disruptive and off-task behaviors for 9%, and aggressive behaviors for 9%.

**Fig. 2:**
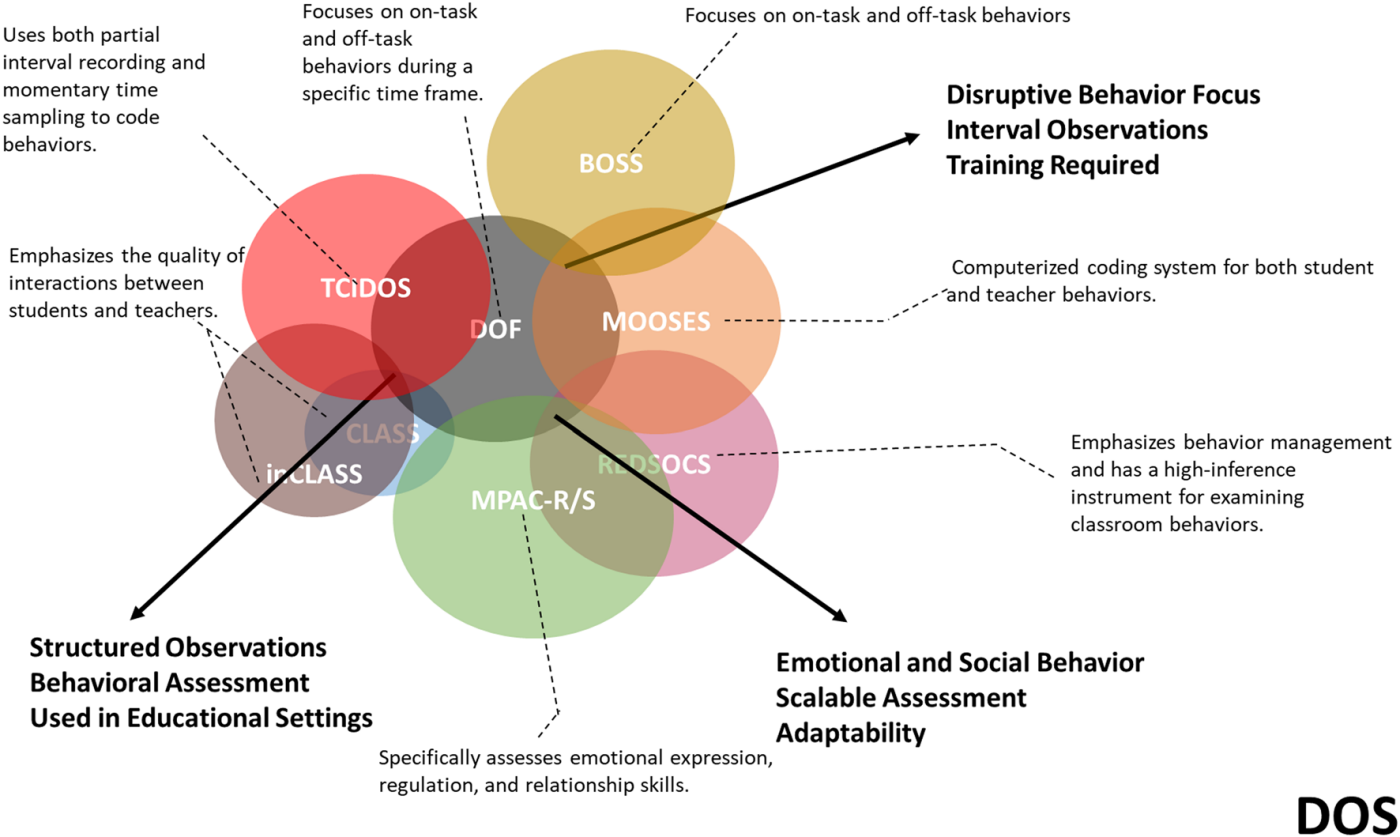
Venn diagram illustrating the overlapping and unique features of various sDOS used in early childhood behavioral assessments. The intersections represent shared features among CLASS and inCLASS, DOF, TCIDOS, MPAC-R/S, MOOSES, REDSOCS, and BOSS, whereas the individual sections highlight the distinct characteristics of each system

For the different sDOSs in the scholarly literature, we found that 38% of the studies used CLASS, 15% used inCLASS, and 12% used DOF. TCIDOS, REDSOCS, and MOOSES were each used by 10% of the studies. In addition, 5% of the studies used other types of instruments. Figure 2 and Table 5 provide a detailed overview of the key features of studies employing DOS, while Table 6 highlights the key features of the 41 studies utilizing the nsDOS tool. The noteworthy results of these studies, organized by instrument, are discussed below.

**Table 5:** Key characteristics of studies using DOS

Authors (Year)	Target Behavior	Sample Characteristics	Observation System
Alamos et al. (2022)	Emotion regulation	N = 767 (49% boys); Age: 4y; Level: Preschool-KG	InCLASS
Bailey et al. (2016)	Cognitive & Social-Emotional Competence	N = 312 (155B, 157G); Age: 3-5y; Level: Preschool	CLASS
Besnard & Letarte (2017)	Social adaptation	N = 180 (50% boys); Age: 4y; Level: Pre-K-KG	CLASS Pre-K
Booren et al. (2012)	Classroom interactions	N = 145 (63B, 82G); Age: 3y; Level: Preschool	InCLASS
Brock & Curby (2014)	Social Competence & Problem Behaviors	N = 2,938 (52% boys); Level: Pre-K; Population: At-risk	CLASS
Bulotsky-Shearer et al. (2014)	Social behavior	N = 304 (50% boys); Age: 3-5y; Level: Preschool; Population: At-risk	CLASS
Chen & Lindo (2018)	On/Off-task behaviors	N = 3 (2B, 1G); Age: 5-6y; Level: KG-1st grade	DOF
Chuang et al. (2020)	Aggressive behaviors	N = 1,817 (52% boys); Level: K-3rd grade; Population: 9% SPED	TCIDOS
Cook et al. (2018)	On/off task behavior	N = 7,419 (48% boys); Age: 6y; Level: KG-2nd grade; Population: General, at-risk, SPED	BOSS
Curby et al. (2021)	Social & learning behaviors	N = 77 (54.24% boys); Age: 4y; Level: Preschool	InCLASS
Denham et al. (2014)	Social-emotional learning behavior	N = 101 (50% boys); Age: 3-4y; Level: Preschool-KG; Population: 56% Head Start	MPAC-R/S
DiPerna et al. (2015)	Social Skills & problem behaviors	N = 432 (46.49% boys); Age: 7y; Level: 2nd grade; Population: GE and SPED	CLASS
Floress et al. (2018)	Externalizing behavior	N = 89; Age: 3-5y; Level: Pre-K-KG; Population: General, at-risk, SPED	REDSOCS; School Observation Coding System
Herndon et al. (2013)	Social-emotional behaviors	N = 308 (51% boys); Age: 3-5y; Level: Preschool	MPAC-R/S
LoCasale-Crouch et al. (2018)	Externalizing behavior	N = 470 (65.7% boys); Age: 4y; Level: Preschool	InCLASS
Meany-Walen & Teeling (2016)	Externalizing behaviors, poor social skills, off-task	N = 5; Level: KG-2nd grade; Population: EBD	DOF
Morris et al. (2013)	Aggressive & disruptive behavior	N = 51 classrooms (15 in each); Age: 4y; Level: Preschool	CLASS; inCLASS
Phillips & Downer (2017)	Engagement & Problem behaviors	N = 116 (51% boys); Age: 4y; Level: Preschool-KG	InCLASS
Reinke et al. (2015)	Aggression	N = 1,818; Level: KG-3rd grade; Population: General and SPED	MOOSES
Shavega et al. (2014)	Behavioral adjustment	N = 320 (50% boys); Age: 4-6y; Level: Preprimary	CLASS Pre-K
Shavega et al. (2019)	Behavioral adjustment	N = 310 (50.32% boys); Age: 5-7y; Level: Preprimary	CLASS
Stanton-Chapman et al. (2014)	Social skills	N = 10 (7B, 3G); Age: 3-5y; Level: Preschool; Population: At-risk	Coding classroom videotapes/MOOSES
Sutherland et al. (2018)	Emotional/behavioral disorders (EBDs)	N = 465 (65.81% boys); Age: 3-5y; Level: Preschool; Population: At-risk	InCLASS, TCIDOS-RV2.1
Swank & Smith-Adcock (2018)	On-task behavior	N = 8; Level: KG-3rd grade; Population: ADHD	DOF
Vujnovic et al. (2014)	Challenging behaviors	N = 21; Age: 4y; Level: Preschool-KG; Population: At-risk and SPED	CLASS
Whittaker et al. (2018)	Disruptive behavior	N = 345 (65% boys); Age: 4y; Level: Preschool; Population: At-risk	CLASS
Williford & Vitiello (2020)	Disruptive behavior	N = 300 (62% boys); Age: 2-5y; Level: Preschool; Population: At-risk	inCLASS
Wolcott & Williford (2015)	Externalizing behavior	N = 360; Age: 3-5y; Level: Preschool; Population: EBD	InCLASS
Zakszeski et al. (2017)	Classroom engagement	N = 24 (54% boys); Age: 3-5y; Level: Pre-K; Population: General, at-risk, SPED	BOSS-EE
Booren et al. (2012)	Classroom behaviors	N = 145 (82 girls); Age: 3-5y; Level: Preschool	inCLASS
Hosterman et al. (2008)	Behavior problems	N = 172 (120 boys); Level: 1st-4th grade; Population: ADHD	BOSS
Bagner & Eyberg (2010)	Externalizing Behavior	N = 68 (67% boys); Age: 3-6y	REDSOCS
Filcheck & McNeil (2004)	Disruptive behavior	N = 30 (86.7% boys); Age: 3-5y	REDSOCS
Pakarinen et al. (2010)	Learning motivation	N = 1268 (655 boys); Age: 6y; Level: KG	CLASS
Rimm-Kaufman & Brock (2009)	Adaptive behaviors	N = 172 (92 boys); Age: 4-6y; Level: KG	CLASS
Curby et al. (2021)	Achievement level	N = 171 (92 boys); Age: 4-6y; Level: KG-1st grade	CLASS
Tiano & McNeil (2006)	Behavior management	N = 16; Population: Head Start	REDSOCS
Tiano et al. (year not provided)	Behavior management	N = 3; Age: 4y; Population: Disruptive behavior	REDSOCS
Conroy et al. (2014a)	Emotional/behavioral disorders	N = 130 (83B, 47G); Age: 3-5y; Level: Preschool; Population: At-risk	TCIDOS
Conroy et al. (2014b)	Engagement & problem behaviors	N = 19 (14B, 5G); Age: 3-5y; Level: Preschool; Population: At-risk	TCIDOS
Denham, Bassett, Mincic, et al. (2012)	Emotional & social behaviors, social problem-solving, self-regulation	N = 275 (50.9% girls); Age: 4y; Level: Preschool; Population: At-risk	MPAC-R/S
Denham, Bassett, Thayer, et al. (2012)	Social-emotional behavior	N = 352 (50.9% boys); Age: 3-4y; Level: Preschool; Population: At-risk	MPAC-R/S
Dillman Taylor et al. (2021)	Disruptive behavior	N = 3; Age: 3-5y; Level: Preschool; Population: At-risk	DOF
Sutherland et al. (2013)	Problem behavior	N = 18; Age: 3-5y; Level: Preschool; Population: At-risk	TCIDOS
Willoughby et al. (2022)	Off-task behavior & disruptive behavior	N = 138 (68% boys); Level: 1st-2nd grade; Population: At-risk	REDSOCS
Gonzales-Ball & Bratton (2019)	Disruptive behavior	N = 20 (15B, 5G); Age: 3y; Population: Head Start, EBD	DOF
Smith et al. (2011)	Externalizing behavior	N = 3 (2B, 1G); Age: 4-5y; Level: Preschool; Population: At-risk	MOOSES

B = Boys, G = Girls; KG = Kindergarten; SPED = Special Education; EBD = Emotional/Behavioral Disorder; GE = General Education; Age and population characteristics presented where reported in original studies

**Table 6:** Key features of studies (N = 41) using nsDOS

Authors (Year)	Target Behavior	Sample Characteristics	Observation Instrument
Alford et al. (2015)	Off-task behavior and Engagement	N = 450 (53.4% girls); Pre-K to 2nd grade; At-risk	PK2 Student Behavior Observation Schedule
Allen & Barber (2015)	Socially appropriate classroom behaviors	N = 20 boys; Age: 5-6y; Level: KG	Developed coded observation form
Benish & Bramlett (2011)	Aggression and peer interactions	N = 3 (2B, 1G); Age: 4y; Level: Preschool; At-risk	POC
Benner et al. (2012)	Externalizing behavior disorders	N = 70 (84.1% boys); Grades K-3; EBD	Stage Observation System
Chen et al. (2011)	Physical aggression	N = 5 (2B, 4G); Age: 3-5y; Level: Preschool; At-risk	Direct observation; ProCoder Digital Version
Conners-Burrow et al. (2017)	Social-emotional behavior	N = 197 preschool teachers; Level: Preschool; 36.7% with disability	Arnett Caregiver Interaction Scale
DiStefano et al. (2013)	Emotional and behavioral risk	N = 1,431 (53.6% boys); Age: 4y; Level: Preschool; At-risk	BESS TRS-P
Donaldson et al. (2017)	Disruptive behavior	N = 12; Level: KG-1st grade	Direct-observation software program
Dunlap et al. (2018)	Challenging behaviors	N = 169 (82% boys); Level: Pre-K; EBD	Direct observation developed by authors
Edwards (2017)	Maladaptive and problem behaviors	N = 3 (1B, 2G); Age: 3-5y; Level: Preschool; EBD	Three formal hour-long running-record observations
Fawley et al. (2020)	Destructive and aggressive Behavior	N = 39 (22B, 17G); Age: 4-5y; Level: Preschool; General and Head start	DPICS 3rd Ed
Floress et al. (2017)	Disruptive behavior	N = 89; Age: 3-5y; Level: Preschool; General, at-risk, SPED	Direct observation using praise recording forms
Fuhs et al. (2013)	Cognitive self-regulation	N = 803 (45.8% girls); Age: 4y; Level: Preschool	Teacher Observation in Preschool
Goble et al. (2016)	Social skills and School readiness	N = 283 (48% girls); Age: 4y; Level: Preschool; At-risk	Brief observation protocol
Gower et al. (2014)	Social-Psychological Adjustment and Physical Aggression	N = 190 (99B, 91G); Age: 4-5y; Level: KG	Naturalistic observation
Greenwood et al. (2018)	Academic Engagement behavior	N = 117 (51% boys); Age: 4-5y; Level: Pre-K; General and SPED	CIRCLE
Hanish et al. (2012)	Aggression	N = 207 (54% boys); Age: 3-5y; Level: Preschool; At-risk	Adaptation of Fagot's interactive coding system
Harvey et al. (2021)	Challenging behaviors	N = 3 boys; Age: 3-4y; Level: Preschool; SPED and EBD	Video-recordings of targeted observation sessions
Hernandez et al. (2016)	Emotional expression, social relationships, school engagement	N = 301 (52% girls); Age: 5y; Level: KG; 36% Head Start	Developed observation tool
Johnson et al. (2016)	Social and Emotional behavior	N = 148 (56% boys); Age: 3-5y; Level: Preschool; At-risk	BEEOS
Luke et al. (2014)	On-task behavior	N = 5 boys; Age: 3-5y; Level: Preschool; SPED	Direct observation
Luo et al. (2017)	Social competence	N = 656; Age: 3-6y; Level: Preschool	Prepublication Version TPOT-P
Metin Aslan (2020)	Aggression and victimization behaviors	N = 105 (61B, 44G); Age: 3-6y; Level: Preschool	Early Childhood Play and Aggression Observation Form
Moffett & Morrison (2020)	Off-task behavior	N = 172 (47% girls); Age: 5y; Level: KG-1st grade	Individualized Student Instruction
Nelson et al. (2017)	Learning engagement behaviors	N = 313 (51.12% girls); Level: Preschool; General, ADHD, EBD, at-risk	MS-CISSAR
Nesbitt et al. (2015)	Disruptive behaviors	N = 1,103 (45.7% girls); Age: 4y; Level: Pre-K; At-risk	Child Observation in Preschool
Prykanowski et al. (2018)	Engagement and Problem Behavior	N = 5 (4B, 1G); Age: 4-5y; Level: Preschool; At-risk	Direct Behavioral Observation
Sallquist et al. (2012)	Social adjustment	N = 166 (54% boys); Age: 4y; Level: Preschool	Fagot's interactive coding system
Schaffner et al. (2016)	Disruptive Behaviors	N = 4; Age: 4y; Level: Preschool; EBD	Preschool Observation Code
Spivak & Farran (2016)	Social competence	N = 60 classrooms (45.6% girls); Level: 1st grade; General	TOP
Ștefan & Miclea (2015)	Social emotional competencies and externalizing problems	N = 3 (2B, 1G); Age: 3-4y; Level: Preschool; At-risk	Paper–pencil method
Stutey et al. (2017)	Externalizing and disruptive behaviors	N = 4 (1B, 3G); Age: 3-5y; Level: Preschool; At-risk	Direct observations
Thomas et al. (2011)	Social Skills and ADHD	N = 137; Age: 3-5y; Level: Preschool; At-risk	Early Screening Profile Social Observation Code
Wood et al. (2011)	Disruptive and challenging behavior	N = 3 boys; Age: 3-5y; Level: Preschool; SPED	Direct observations
Wright et al. (2022)	Social behavior	N = 325 (51% boys); Level: 1st grade; At-risk	Direct observation
Zaghlawan & Ostrosky (2011)	Social skills and challenging behaviors	N = 15–20 per circle time; Level: Head Start	Observational Coding System
Öneren Sendil & Erden (2019)	Peer relationship problems	N = 46 (22B, 24G); Age: 4-6y; Level: Preschool; No disability	79 h qualitative classroom observation
Tucker et al. (2017)	Social-emotional skills, behavioral regulation, problem-solving	N = 206; Age: 3-4y; Level: Preschool	Teaching Pyramid Observation Tool
Morgan et al. (2018)	Social, communication, emotional regulation	N = 197 (81.2% boys); Age: 6y; Level: KG-2nd grade; ASD	Classroom Measure of Active Engagement
Snyder et al. (2011)	Conduct problems	N = 136 (57% boys); Age: 4y; Level: Preschool;At-risk	Classroom Interaction Coding system

B = Boys; G = Girls; KG = Kindergarten; SPED = Special Education; EBD = Emotional/Behavioral Disorder; ASD = Autism Spectrum Disorder; MS-CISSAR = Mainstream Version-Code for Instructional Structure and Student Academic Response; CIRCLE = Code for Interactive Recording of Children's Learning Environments

### CLASS and iCLASS

Studies utilizing the CLASS and inCLASS have elucidated the impact of teacher support, classroom interventions, settings, and interactions on children’s socioemotional and behavioral regulation. Bailey et al. (2016) reported that teacher emotional and organizational support positively predicts children's classroom engagement, highlighting the importance of supportive environments [8]. DiPerna et al. (2015) showed that a social skills intervention improved student behavior, especially for lower-skilled children, demonstrating the benefits of targeted skill building [26]. Morris et al. (2013) revealed that an intervention strengthened teachers' behavior management and cultivated more positive emotional classroom climates [56]. Regarding specific settings, Booren et al. (2012) found that children displayed more positive interactions with teachers in structured activities than in child-directed activities [12]. Alamos et al. (2022) emphasized preschool classrooms as pivotal contexts for emotional development and socialization([2]). Several studies have revealed complex associations between teacher practices and child behavior. For instance, Williford and Vitiello (2020) demonstrated bidirectional links between teacher interactions and children's disruptive behaviors [78]. Wolcott and Williford (2015) highlighted the value of using observational and rating methods together to comprehensively evaluate child externalizing behaviors [79]. Phillips and Downer (2017) revealed an intricate interplay among classroom factors, teaching experience, and perceptions of child engagement [60].

#### REDSOCS

Studies utilizing the REDSOCS have investigated various aspects of children's behavior and classroom interactions. A focus on EBD/ADHD was also evident in the research of Fawley et al. (2020), who utilized the REDSOCS version of Ginn et al. (2009) to further investigate the nuances of such behaviors in the pre-elementary setting [31[. Floress et al. (2018) also used REDSOCS to study children with EBD/ADHD, emphasizing the interaction between these behaviors and classroom interactions [33]. Collectively, these studies underscore the utility of the REDSOCS instrument in capturing various behavioral patterns in children, with a particular emphasis on those with EBD and ADHD, and its implications for classroom dynamics and teacher-student interactions.

#### DOF

Studies employing DOF have provided insights into child behavior across diverse educational settings and populations. In elementary contexts, DOF has been utilized to examine general classroom behaviors, without a specific focus on disabilities [16, 36]. However, other studies have concentrated directly on the utility of DOFs for capturing behavior in children with EBD and ADHD [52, 74]. These studies demonstrate the versatility of DOF in elementary settings for both broad and targeted behavioral assessments.

Expanding beyond elementary grades, Taylor et al. (2021) applied DOF specifically to preschool children identified as at-risk, underscoring its value in early observation and intervention during preschool [25]. Overall, DOF research highlights the adaptability of the instrument across educational settings from preschool to elementary school, diverse student populations from general education to special needs, and a range of assessment purposes from broad observations to diagnoses of specific disorders such as ADHD. The findings reveal that DOF is an efficacious tool for capturing the multidimensional spectrum of children’s classroom behaviors to inform supportive practices.

### MOOSES, MPAC-R/, BOSS, and TCIDOS

Other specialized direct observation tools, such as MOOSES, MPAC-R/, BOSS, and TCIDOS, have also provided valuable insights into child behaviors and teacher-student interactions. MOOSES has been utilized in both preschools [67, 69] and elementary setings [62] to observe behaviors ispecial-educationcial education populations. The Minnesota Preschool Affect Checklist, Revised and Shortened.(MPAC-R/S) has been applied in preschool contexts to capture emotional and behavioral patterns, including in children with EBD/ADHD and in head start programs [24, 41]. The TCIDOS has been used in multiple studies focusing on at-risk preschool populations [21, 73]. BOSS has provided insights into ADHD and EBD behavioral profiles, specifically in elementary classrooms [22].

#### Key findings of studies using nsDOS

Several studies have used the nsDOS to assess child and teacher behaviors in classroom settings with significant diversity and rigor. A recent study by Conners-Burrow et al. (2017) utilized the Arnett Caregiver Interaction Scale (CIS) to observe teacher–child interactions [20]. The CIS uses a rating scale to evaluate behaviors, including teacher sensitivity and ineffective actions such as harshness or lack of involvement. Classroom structure was assessed through observations of schedules, transitions, and related teacher practices. Wood et al. (2011) conducted direct observations using interval recordings to code behaviors [80]. Ştefan and Miclea (2015) employed multiple-baseline design and teacher rating scales such as the Social Competence Screening for Preschoolers-Teacher Form (SCS-T) and the Emotion Competence Screening for Preschoolers-Teacher Form (ECS-T) to evaluate social-emotional competencies [70]. SCS-T contains items on compliance, interpersonal skills, and prosocial actions. ECS-T measures emotional understanding, expression, and regulation. Both scales demonstrated strong internal consistency. The authors also utilized a subscale of the Social Competence and Behavior Evaluation Scale.

The Autism Diagnostic Observation Schedule and Classroom Measure of Active Engagement were used to confirm autism diagnoses and quantify engagement via video coding (Morgan et al., 2018). The Mainstream Code for Instructional Structure and Student Academic Response (MS-CISSAR) enables detailed sampling of behaviors and contexts through computer-assisted coding [57]. This demonstrated its validity in predicting achievement. Other studies measured social skills, emotions, aggression, etc., using interval recording, time sampling, or validated instruments such as the Teaching Pyramid Observation Tool (TPOT) [76]. Reliability was established through intercoder checks of live observations or videos [65, 69].

Zakszeski et al. (2017) compared different interval durations for momentary time sampling (MTS) in measuring young children's engagement and reported that shorter intervals better approximated continuous duration recording [81]. Fawley et al. (2020) used coding systems such as the Dyadic Parent–Child Interaction Coding System (DPICS)—Third Edition and the Revised Edition of the School Observation Coding System (REDSOCS), with 2-min observation samples conducted multiple times per week to evaluate teacher–child interactions [31]. Greenwood et al. (2017) employed an MTS with 15 s intervals and the Code for Interactive Recording of Children’s Learning Environments (CIRCLE) observation tool to record children's learning environments [37]. Chen et al. (2011) collected biweekly naturalistic observations of children's aggressive and rejection behaviors using the specialized ProCoder Digital Version software. Across these studies, direct observation methods were tailored to assess specific classroom behaviors, and reliability was checked through interobserver assessments [15]. While specific tools and intervals varied, the approaches shared a commitment to gathering rigorous observational data on teacher and student actions through systematic coding protocols.

Chuang et al. (2020) observed aggressive behaviors using the Student–Teacher Classroom Interaction Observation code after extensive observer training to ensure reliability [17]. DiStefano et al. (2013) developed the Behavior Assessment System for Children and reported strong psychometric properties, including interrater reliability [27]. Donaldson et al. (2017) assessed disruptive behaviors through direct observation with instant data software, defining categories such as out-of-seat actions [28]. In addition, Dunlap et al. (2018) measured student engagement time using MTS procedures, which have demonstrated reliability in past research. In these studies, standardized observation tools, specialized software programs, and customized recording procedures were used to reliably evaluate various classroom behaviors through systematic direct observation protocols and rigorous training.

Standardized observation tools, such as Teacher Observation in Preschools (TOP) and Child Observation in Preschools (COP), have been used to systematically assess teacher and student behaviors in preschool classrooms [34, 68]. The TOP framework focuses on quantifying teachers' actions, whereas the COP system records individual child behaviors that can be aggregated into classroom-level metrics. These protocols use time-sampling procedures, in which each participant is observed briefly (3–5 s) and then coded across categorical frameworks. For example, COP includes codes for verbalizations, peer interactions, task focus, and involvement. The observers cycle by coding each participant in a process termed a "sweep" before starting the next round of observations. Studies have reported sound interrater reliability for these tools based on training observers to criterion levels through extensive practice and anchoring to expert coders. Fuhs et al. and Spivak & Farran reported kappa values ranging from.82 to.87 for the TOP and COP. Nesbitt, Farran, & Fuhs also successfully used COP and reported adequate interrater agreement for engagement codes[58]. Overall, systematic protocols such as the TOP and COP allow rigorous quantification of teacher practices and child behaviors through time sampling, multiple observation sweeps, and training procedures, which yield reliable observational metrics in preschool settings.

Floress et al. conducted direct classroom observations to record teachers' use of praise across general education, at-risk, and special education settings[32]. They used customized praise recording forms to capture the key dimensions of praise delivery. Each form allowed observers to tally instances of behavior-specific praise and general praise across individual, small-group, and large-group delivery methods. This approach allowed fine-grained quantification of the praise type and recipient. The praise recording form developed by Floress et al. demonstrated the utility of customized protocols for the direct observation of teacher practices based on target constructs. The ability to reliably distinguish praise types and recipient groups illustrates the value of aligning observation tools with research questions when gathering classroom observational data [32].

Several studies have utilized time sampling procedures to conduct naturalistic observations of student behavior in school settings. Goble et al. used a validated 10-s observation protocol to quantify social interactions indoors and outdoors multiple times per week [35]. Gower et al. also employed time sampling to record aggression during free play periods longer than 8 weeks, with intercoder reliability checks. Hanish et al. adapted an interactive coding system for 10-s interval observations of behaviors and peer reactions across play sessions [38]. Harvey et al. gathered video recordings of classroom transitions once weekly and coded the target behaviors [39]. These approaches collect observational data in authentic contexts, capitalizing on the benefits of time sampling to capture behavioral snippets that can be aggregated to provide robust metrics. Goble et al. demonstrated how even 10-s snapshots layered over time can reliably indicate social engagement [35]. Recording frequencies of aggression using similar brief intervals illustrate how time sampling lends itself to quantifying discrete behaviors. Video recording transitions were used in accordance with Harvey et al., who used targeted footage to evaluate the behavior of interest [39]. Harvey, Dunlap, and McKay video-recorded targeted sessions, such as transitions between activities, to sample student challenges while minimizing disruption[39]. Coding videos offline also reduced the need for live observers. The ability to define observation duration and settings based on the behaviors of interest, as shown in Harvey et al.'s study, highlights the flexibility of direct observation. Overall, these investigations exemplify a range of techniques for gathering observational data through purposeful time sampling, adaptation of established tools, video recording, and coding choices tailored to research questions. Combined with inter-observer reliability checks, such customized applications can expand the potential of direct observation in educational research.

Hernández et al. developed a coding system to rate engagement during academic activities using 30-s intervals [40]. Reliability was established through intercoder assessments of prerecorded and live observations. Johnson et al. (2016) employed the Behavioral Emotions and Expression Observation System (BEEOS) to capture emotional and behavioral constructs during classroom play activities [45]. Observations occurred over 8-min periods with ratings at 15-s intervals guided by an audio cue. Inter-rater reliability was checked using two observer codes for a subset of students. The use of audio cues introduced by Johnson et al. demonstrates how even basic technology can facilitate precise interval-based sampling [45]. Reliability assessments using intermittent dual coding further bolstered the credibility of the data gathered using these focused observational methods.

Allen and Barber used an interval-based coding system to quantify on- and off-task actions during 30-min observation sessions [5]. Luo et al. employed the Teaching Pyramid Observation Tool for Preschool Classrooms (TPOT-P) to evaluate teaching practices, concerning behaviors, and responses across 2-h naturalistic observations [50]. The TPOT-P contains key indicators organized into coding categories. Moffett and Morrison leveraged the Individualized Student Instruction system's ability to code activity types, instructional contexts, and attention direction [54]. These studies demonstrate the range of approaches for gathering observational data, from tailored coding frameworks to standardized instruments such as the TPOT-P. Strategies such as Aslan's mixed methods illustrate how qualitative and quantitative techniques can be integrated into a comprehensive perspective [53]. Reliability assessments were consistently incorporated, although the methods varied across live observations, video coding, and instrument design.

#### Features of nsDOS

**Fig. 3:**
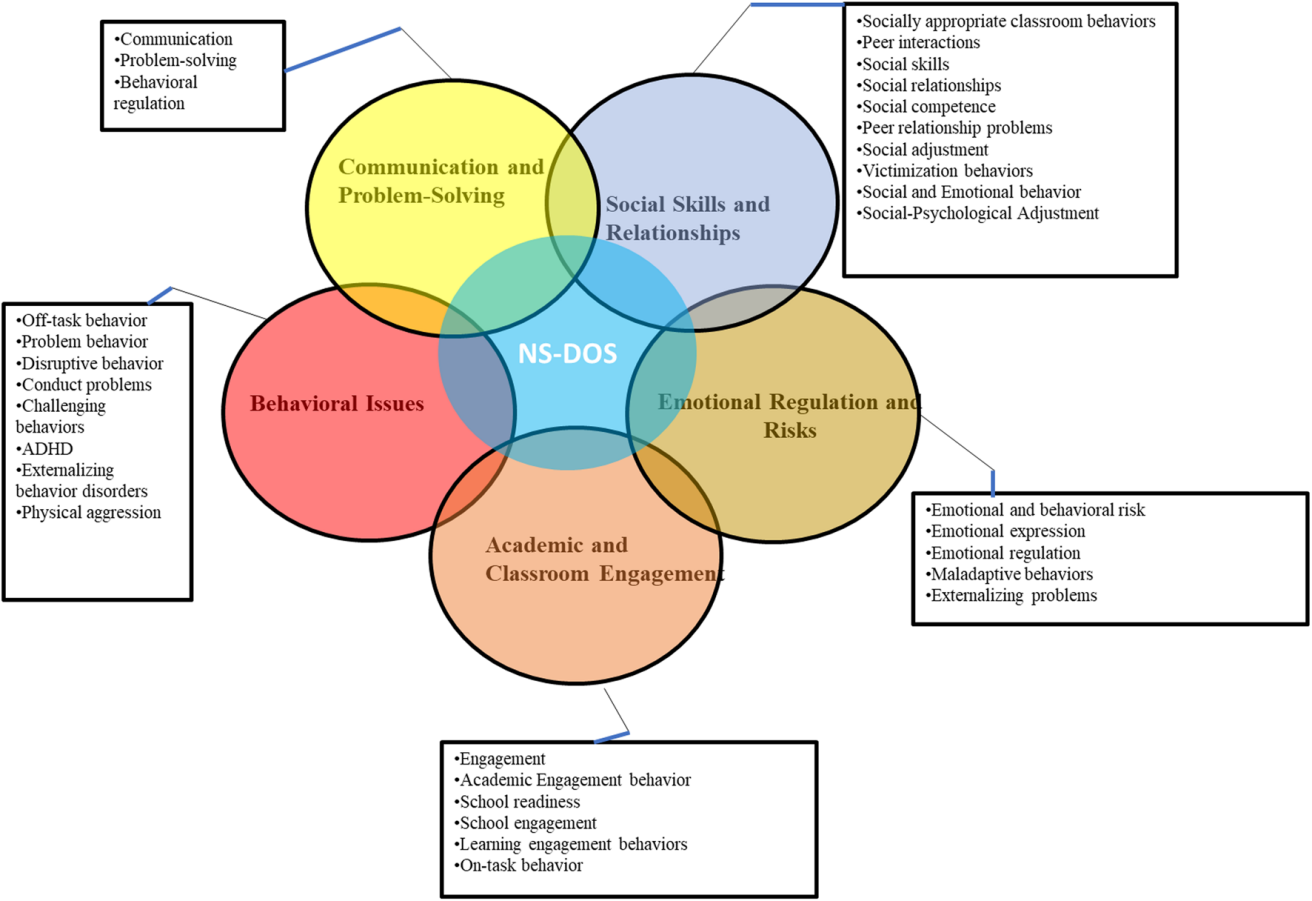
Diagram illustrating the categories of nsDOS in Child Behavior Studies. The diagram shows the primary focus areas: behavioral issues, emotional regulation and risks, social skills and relationships, social and emotional behavior, sociopsychological adjustment, academic and classroom engagement, and communication and problem-solving. Each focus area contained specific behaviors or issues commonly observed, revealing the multidimensional nature of child behavior and the interconnectedness of these categories

The above studies clearly indicate that the landscape of nsDOS used in the literature is rich and diverse, covering a wide array of themes that address the complex needs and behaviors of students in classroom settings (Fig. 3). A notable trend was the utilization of custom observation schedules, such as the "PK2 Student Behavior Observation Schedule," which is often applied to evaluate off-task behaviors and engagement. Some studies have also focused on problem behaviors using extensive qualitative observations lasting up to 73 h. Additionally, a subset of related research has employed uniquely developed coded observation forms or paper–pencil methods, often aimed at assessing socially appropriate classroom behaviors and social-emotional competencies. These methods have often been employed in studies focusing on children aged to 5–6 years, particularly in kindergarten settings. Another category of studies utilized specialized software programs for direct observation. These tools, such as the "Direct Observation; ProCoder Digital Version software program," were mainly used to focus on aggressive and disruptive behaviors, often in preschool settings with children classified as 'at-risk.’ Specialized coding systems, such as the “DPICS 3rd Ed” and [CIRCLE], have also been used in a range of studies. From understanding behavioral issues to promoting social skills and academic engagement, these studies offer invaluable insights into the factors that contribute to a successful educational experience. A more detailed exploration of these themes is shown in Fig. 3.

### Behavioral issues and emotional regulation

Studies in this category have focused on various forms of disruptive and off-task behavior. Alford et al. (2015) examined off-task behavior and engagement, highlighting the importance of keeping students focused on the classroom([3]). Floress et al. and Nesbitt et al. delved into disruptive behaviors, emphasizing the need for effective classroom management strategies [32, 58]. Benner et al. and Chen et al. explored externalizing behavior disorders and physical aggression, respectively, shedding light on more severe behavioral issues that require specialized interventions [10, 14]. This theme also encompasses studies that examine emotional and behavioral risks and how they manifest in educational settings. For example, DiStefano et al. focused on emotional and behavioral risks. These studies underscore the importance of early identification and intervention for emotional and behavioral issues that can impede academic success [27].

### Social skills and relationships

Research in this category often explores the social dynamics within educational settings. Allen and Barber studied socially appropriate classroom behaviors, whereas Goble et al. examined social skills and school readiness [5, 35]. Benish and Bramlett focused on aggression and peer interactions, highlighting the complex social challenges that children face in school settings [9].

### Academic and classroom engagement

Studies on this topic aim to understand how engagement affects academic outcomes. Greenwood et al. (2018) examined academic engagement behaviors, whereas Nelson et al. studied learning engagement behaviors [37, 57]. These studies suggest that engagement is a critical factor in academic success and should be a focal point in educational interventions.

### Communication and problem solving

This category includes studies that explore the skills necessary for effective communication and problem solving within the educational context. Morgan et al. focused on social, communication, and emotional regulation, whereas Tucker et al. examined social-emotional skills, behavioral regulation, and problem solving [55, 76].

## Discussion

In the dynamically evolving field of educational research, DOS has become a vital tool for objectively assessing children's behaviors in educational settings. This systematic review included 88 studies focusing on both standardized data and nsDOS scores in early childhood education. These findings suggest that while the sDOS offers a reliable method for generalized behavioral assessment, the nsDOS is also widely and effectively used because it provides the flexibility required for more targeted behavioral evaluations.

Standardized instruments, such as the CLASS and inCLASS, are the mainstays, appearing in nearly 60% of the studies based on sDOS. These tools are widely recognized for their reliability and are commonly used to assess a range of behaviors, from emotional regulation to social-emotional competence and classroom interactions ([2]). In contrast, nsDOSs exhibit rich diversity, with custom observation schedules and specialized coding systems being particularly prevalent. These nonstandardized tools often fill the gaps left by their standardized counterparts, capturing specific behaviors that require a more tailored approach.

While standardized tools often concentrate on emotional regulation and social skills, the nsDOS offers a more targeted approach, focusing on specific behaviors such as off-task behavior, aggression, and academic engagement. Given the complementary strengths of both types of DOS, educational institutions and policymakers should adopt a multimethod approach for a more comprehensive behavioral assessment. This review also highlights various studies that have employed direct observation techniques to assess both child and teacher behavior in classroom settings. These studies used a range of methods, from MTSs to specialized coding systems, to capture specific classroom behaviors. Despite the diversity in tools and intervals, these studies share a commitment to rigorous data collection and reliability through interobserver assessments.

For instance, Zakszeski et al. compared different interval durations for MTS in measuring young children's engagement and found that shorter intervals better approximated continuous duration recordings [81]. Fawley et al. used coding systems such as DPICS and REDSOCS with 2-min observation samples conducted multiple times per week to evaluate teacher‒child interactions [31]. Greenwood et al. employed MTSs with 15 s intervals and the CIRCLE observation tool to record children's learning environments [37]. Floress et al. and Cook et al. used REDSOCS and variations of the BOSS system, respectively, to code student on-task behaviors [22, 33]. Chen et al. collected biweekly naturalistic observations of children’s aggression and rejection behaviors using specialized software [14]. Across these studies, the DOS was tailored to assess specific classroom behaviors, and reliability was checked through interobserver assessments. While specific tools and intervals varied, the approaches shared a commitment to gathering rigorous observational data on teacher and student actions through systematic coding protocols.

### Significance of this review for practice and research

This systematic review highlights several important insights, with implications for research and practice in early childhood education. This review emphasizes the utility of both standardized and non-standardized DOS for capturing a range of child behaviors. Standardized instruments, such as the CLASS and inCLASS, which were used in approximately 59.6% of the studies, offer reliability for general behavioral assessments. On the other hand, the nonstandardized DOS provides flexibility for targeted evaluations, especially for at-risk populations, which comprised 48.8% of the studies using the nonstandardized DOS. This finding suggested that a combined approach using both types of DOS may offer a more comprehensive framework for behavioral assessment. Another key finding was the focus on high-need and at-risk populations, particularly those diagnosed with EBD or ADHD. This focus highlights the need for specialized educational interventions and professional development for educators to manage these specific behavioral challenges. The review also identified methodological gaps that need attention, such as limitations related to sample sizes and potential observer bias. A notable gap in current DOS implementation is the lack of integrated post-assessment support packages, particularly for children at risk or with special needs. While systems such as CLASS provide general quality improvement suggestions, none of the reviewed DOS offer comprehensive, structured intervention guidelines following assessment. This represents a significant opportunity for DOS development, particularly given the increasing need for evidence-based intervention support in early childhood settings. This calls for more rigorous research protocols in the future. Additionally, the review revealed that future research should explore a more representative and multicenter approach to behavioral assessment to streamline and harmonize the use of DOS in early education.

### Limitations

Several methodological constraints should be considered when interpreting the findings of this systematic review. Our primary limitation stems from the scope parameters, which focused exclusively on DOS in early childhood behavioral research, without incorporating a comprehensive analysis of IOA metrics across studies. Although IOA constitutes a fundamental quality indicator in observational research [77], the systematic evaluation of IOA methodologies exceeded our analytical framework. This review's search methodology has additional limitations. Our protocol restricted inclusion to English-language peer-reviewed publications indexed in specified databases (PsycINFO, ERIC, and Web of Science), potentially excluding pertinent research from non-Anglophone scholarly communities. Although this approach aligns with established systematic review protocols, it may have introduced language and publication bias (Higgins et al., 2019). Furthermore, despite implementing a comprehensive Boolean search strategy, the heterogeneous nature of behavioral observation terminology may have resulted in the inadvertent omissions of relevant studies. For nonstandardized direct observation systems (nsDOS), we employed a temporal delimitation of the past decade (2014–2024). While this criterion enhanced the contemporary relevance of our findings, it potentially excluded seminal methodological contributions that could have provided valuable historical context. The manual screening process, although conducted independently by multiple reviewers with acceptable inter-rater reliability (κ = 0.85), remains susceptible to human error. Our analytical framework prioritized methodological characteristics and empirical outcomes of DOS implementations, potentially underrepresenting the pragmatic challenges associated with field applications. The exclusion of gray literature and implementation reports may have limited our understanding of real-world applications and contextual adaptations of these observational systems. Future systematic reviews could address these limitations through expanded linguistic inclusion criteria, broader temporal parameters, and systematic analysis of IOA metrics. Additionally, incorporating mixed methods approaches could better capture the complexity of DOS implementation in naturalistic settings. These methodological refinements would contribute to a more comprehensive understanding of DOSs in early childhood behavioral research.

## Conclusions

This systematic review of nsDOS and sDOS tools examines methodologies for assessing children's behavior in early childhood education. The analysis revealed CLASS/inCLASS as a frequently used instrument, while also highlighting a diversity of other tools employed to study classroom interactions. Our findings suggest potential associations between teacher practices, classroom climate, and peer dynamics on developmental outcomes, though causal relationships cannot be definitively established from the reviewed studies. The results indicate that DOS tools can capture various emotional, attentional, and behavioral constructs, with particular utility observed in studies involving at-risk or special needs children. Several limitations must be acknowledged: observer bias remains a significant concern, potentially affecting data quality; the influence of extraneous variables in naturalistic settings was often inadequately controlled; and methodological heterogeneity across studies limited our ability to draw robust comparative conclusions. Future research would benefit from standardized protocols to enhance assessment consistency and facilitate cross-study comparisons, alongside more rigorous control of confounding variables. While this review provides a synthesis of current observational research practices in early childhood settings, it also underscores the need for more methodologically robust studies to establish a stronger evidence base for informing supportive practices in early childhood development.

## Supplementary Information

Below is the link to the electronic supplementary material.

Supplementary file1 (DOCX 19 KB)

## Data Availability

No datasets were generated or analysed during the current study.
