# Supplementary material for: Direct observation systems for child behavior assessment in early childhood education: a systematic literature review
Source: Discov Ment Health. 2025 Feb 24;5(1):21. doi: 10.1007/s44192-025-00139-z (PMC11850679; doi:10.1007/s44192-025-00139-z)
Supplement: Supplementary file 1 — Supplementary file1 (DOCX 19 KB) [file 44192_2025_139_MOESM1_ESM.docx]

Appendix 1

Features of sDOS

BOSS

Behavioral Observation of Students in Schools (BOSS) is grounded in applied behavior analysis, which utilizes the principles of learning theory to objectively measure observable behaviors. Applied behavior analysis informed the BOSS's use of structured observation techniques to collect frequency and duration data on student behaviors of interest (Shapiro, 2004). This framework drives BOSS's focus on directly capturing classroom behaviors to identify the environmental factors that influence them. It measures behaviors such as active working, listening, passive off-task, motor off-task, and verbal disruption. BOSS combines interval recording, which tracks behavior durations, and time sampling, which notes point-in-time occurrences, to provide comprehensive data. The BOSS has strong psychometric properties. Inter-rater reliability ranges from 0.87 to 0.96 across studies (Volpe et al. 2005). Validation analyses indicate that BOSS on-task categories converge with other engagement measures while differentiating between the normal and ADHD groups (Volpe et al. 2006).

CLASS

The Classroom Assessment Scoring System (CLASS) is grounded in attachment theory, which emphasizes the importance of positive teacher-child relationships that make children feel safe, understood, and valued (La Paro et al., 2012). Drawing on this framework, CLASS focuses on observing the emotional and instructional support teachers provide through their language, responsiveness, affection, and modeling. CLASS has strong psychometric properties. It shows high internal consistency, with values ranging from.79 to.94 across studies (Hamre et al. 2013). Validation research also supports links between CLASS scores and positive teacher-child interactions, child development outcomes, and learning environment quality (Dawson & Lauricella, 2021). Originally designed for professional development applications aimed at improving teacher-student interactions, CLASS examines ten dimensions of classroom processes: positive climate, negative climate, teacher sensitivity, standards for student perspectives, behavior management, productivity, instructional learning formats, concept development, and quality of feedback (La Paro et al., 2012). CLASS employs interval recording with 20 min of observation followed by 10 min of coding. This provides rich rate and duration data but can miss brief events occurring between intervals (Rapp et al., 2008). In contrast, some systems, such as BOSS, supplement CLASS's interval recording with time sampling, which captures more discrete behaviors but offers less data on behavior length. Using both interval and time sampling approaches allows for comprehensive observational data collection.

inCLASS

The Individualized Classroom Assessment Scoring System (CLASS) is also derived from attachment theory and its classroom-focused complement, the ecological systems theory. This dual framework recognizes that secure teacher relationships and classroom support foster child development (Downer et al. 2010). inCLASS's observation categories emanate from these theories' recognition that teacher, peer, and activity engagement promote student outcomes through positive environmental interactions. The Individualized Classroom Assessment Scoring System (inCLASS) demonstrates an inter-rater reliability of.71-.95 across domains (Downer et al., 2010). It exhibits the expected associations with emotional regulation abilities (Jeon et al., 2014). inCLASS stemmed from earlier CLASS versions that focused on individual child interactions versus the overall classroom climate (Downer et al., 2010). It includes dimensions such as positive engagement with teachers, peer sociability, and task behavior.

MOOSES, MPAC-R/S, REDSOCS, and TCIDOS

The Multi-Option Observation System for Experimental Studies (MOOSES) is grounded in principles of applied behavior analysis and experimental methodology (Tapp, 2004). MOOSES draws on behavioral analysis to objectively measure target behaviors and environmental variables in controlled settings. Its flexible coding aligns with experimental design tenets on adapting measurements to research questions, enabling hypothesis testing through customizable recording. MOOSES demonstrates exceptional adaptability to diverse research contexts by enabling customized coding and analysis of observed behaviors (Tapp, 2004). This versatility allows seamless integration into various designs while ensuring data accuracy and relevance. MOOSES also provides time-efficient coding with detailed timestamping and advanced analytic features such as lag sequential analysis. Reliability ranges from.71 to.99 across studies (McWilliam, 1999).

The Motivation Assessment Scale (MPAC-R/S) is rooted in functional behavior assessment, which examines behavioral antecedents and consequences (Boone 1992). By categorizing motivations, the MPAC-R/S provides functional assessment data to identify reinforcers and facilitate interventions. Inter-rater reliability averages.89, and validation supports links between MPAC-R/S and behavior function (Singh et al., 2011). The Researcher-Educator Collaboration for Developing Observation Coding Systems (REDSOCS) stems from participatory action research principles on collaborative inquiry (Casler et al., 2020). This drives the REDSOCS framework, which integrates researcher and educator expertise to create observation tools tailored to unique research or classroom needs. The inter-rater reliability exceeds.80 across studies. The Teacher-Child Interaction Direct Observation System (TCIDOS) is derived from ecological systems theory, situating child development within interconnected environments (McWilliam, 1999). TCIDOS focuses on the teacher-student microsystem, informing observations of their bidirectional impacts. It demonstrates convergent validity with the CLASS (Curby et al., 2011) and examines dimensions such as verbal instruction, scaffolding, and behavior regulation.
